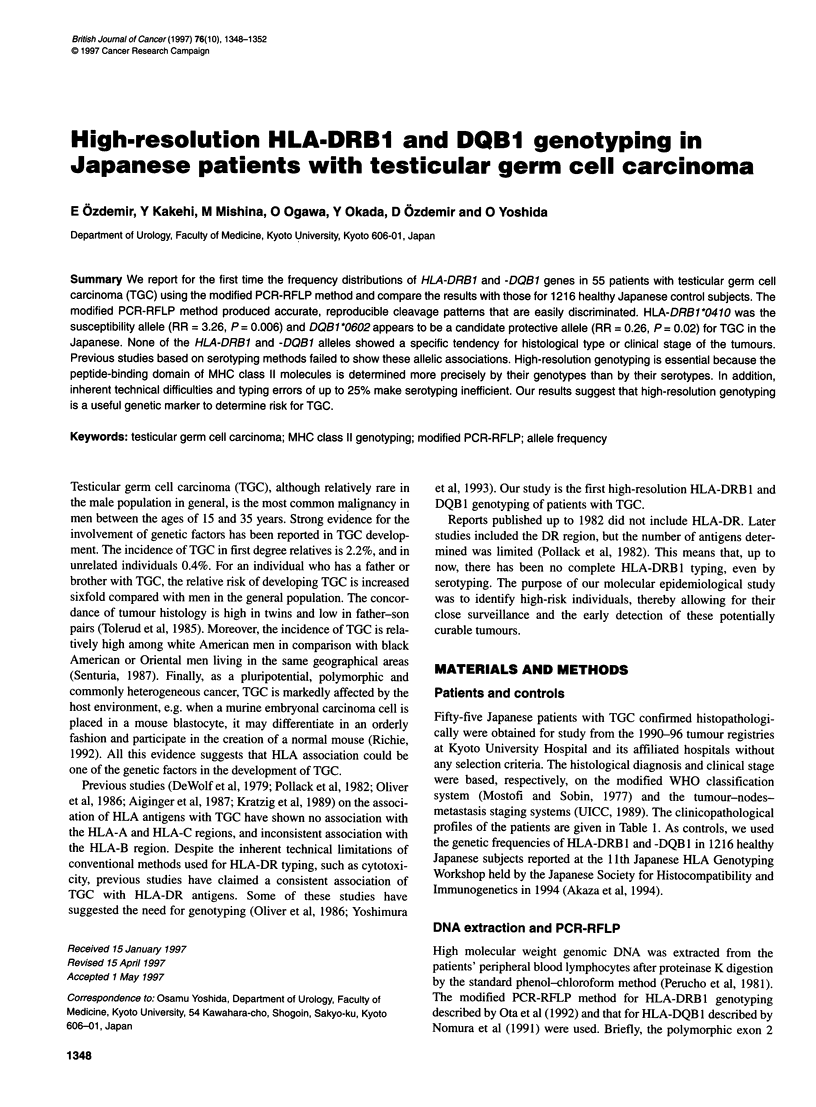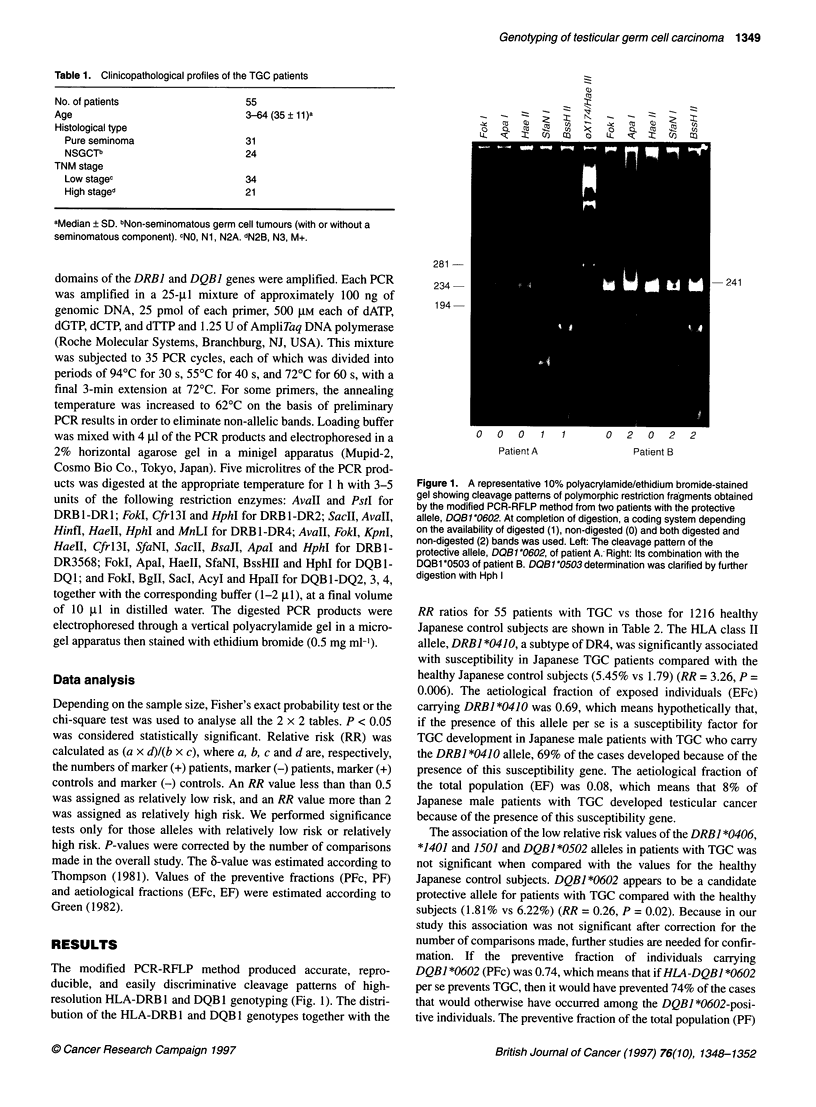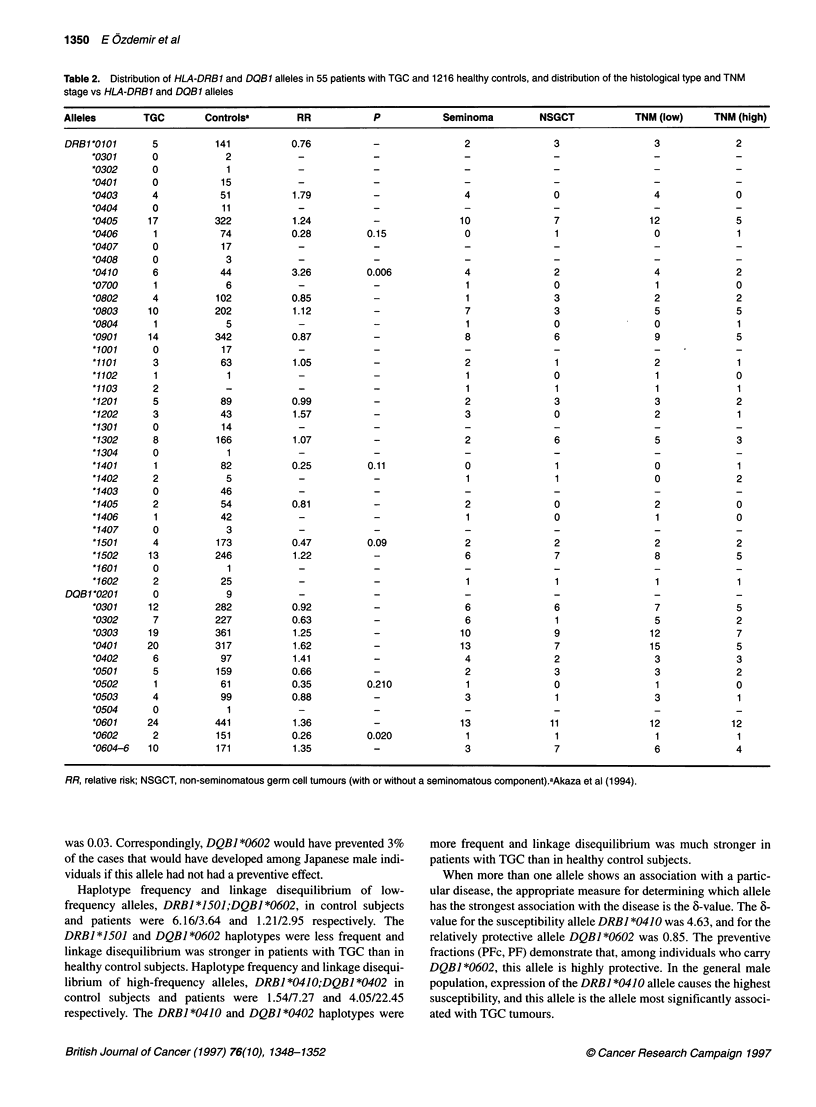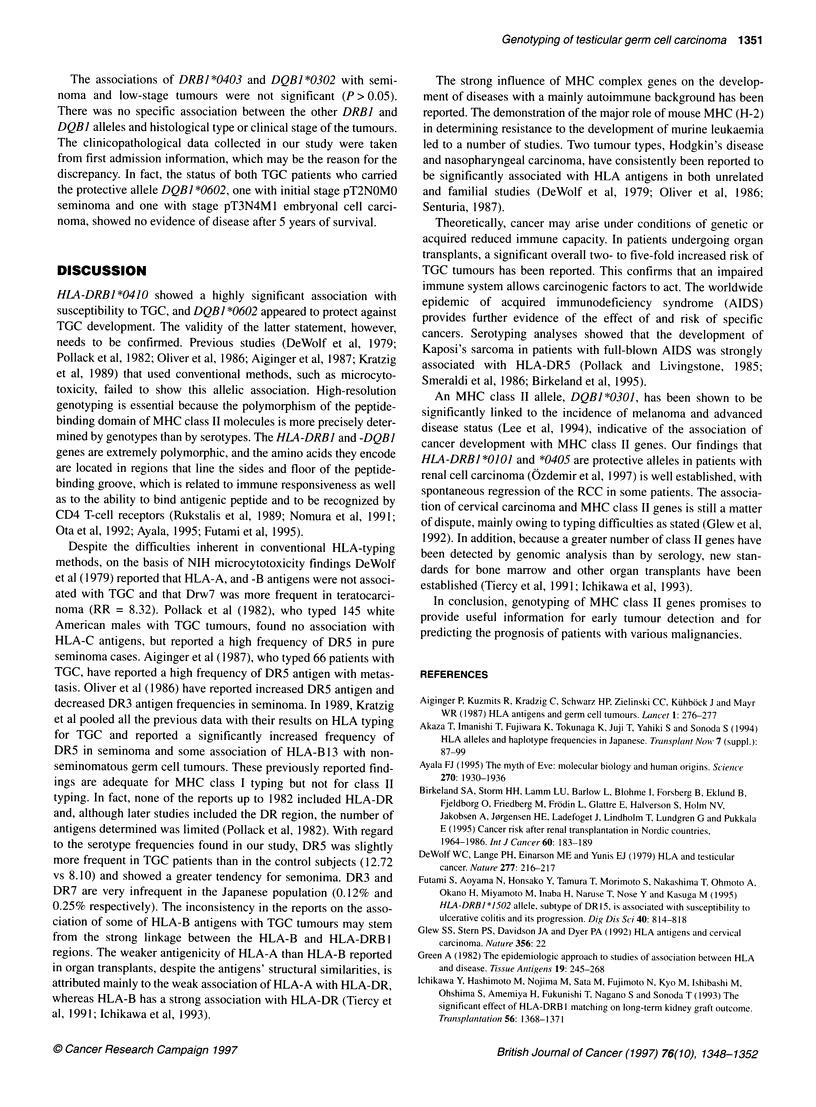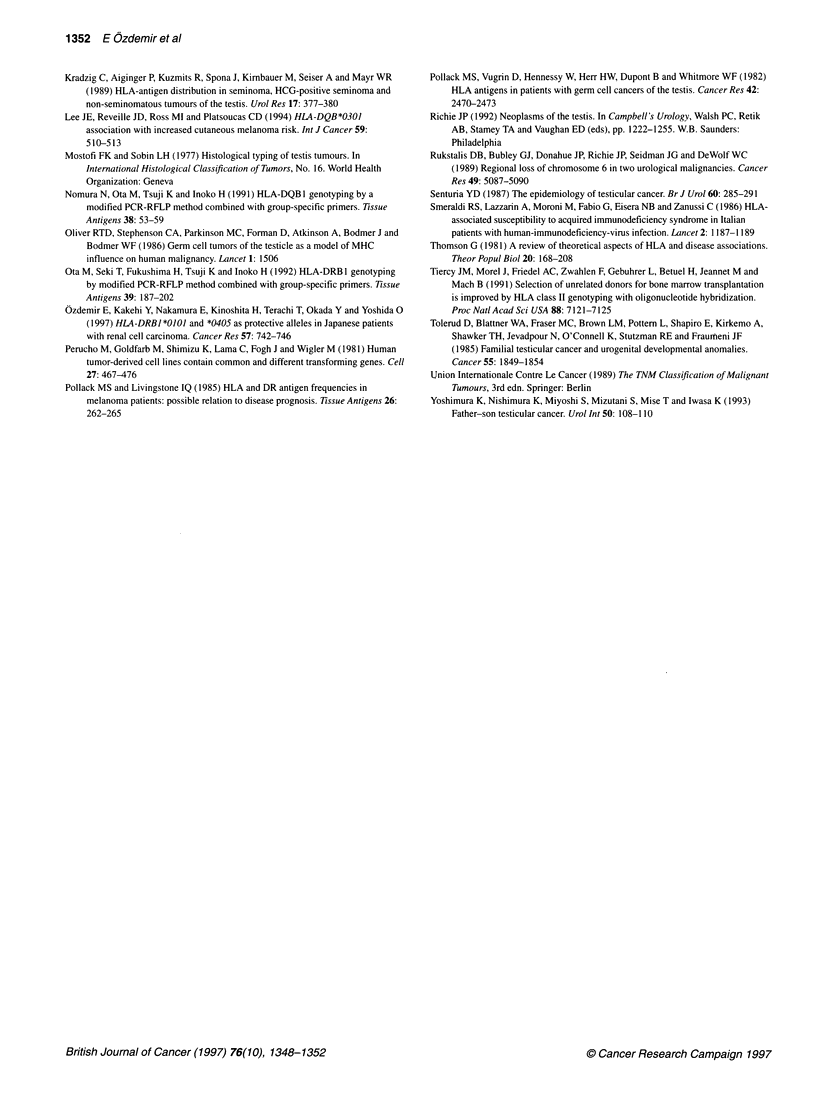# High-resolution HLA-DRB1 and DQB1 genotyping in Japanese patients with testicular germ cell carcinoma.

**DOI:** 10.1038/bjc.1997.559

**Published:** 1997

**Authors:** E. Ozdemir, Y. Kakehi, M. Mishina, O. Ogawa, Y. Okada, D. Ozdemir, O. Yoshida

**Affiliations:** Department of Urology, Faculty of Medicine, Kyoto University, Japan.

## Abstract

**Images:**


British Journal of Cancer (1997) 76(10), 1348–1352
© 1997 Cancer Research Campaign

### High-resolution HLA-DRB1 and DQB1 genotyping in Japanese patients with testicular germ cell carcinoma

**E Özdemir, Y Kakehi, M Mishina, O Ogawa, Y Okada, D Özdemir and O Yoshida**

Department of Urology, Faculty of Medicine, Kyoto University, Kyoto 606-01, Japan

**Summary** We report for the first time the frequency distributions of *HLA-DRB1* and *-DQB1* genes in 55 patients with testicular germ cell carcinoma (TGC) using the modified PCR-RFLP method and compare the results with those for 1216 healthy Japanese control subjects. The modified PCR-RFLP method produced accurate, reproducible cleavage patterns that are easily discriminated. HLA-*DRB1**0410* was the susceptibility allele (RR = 3.26, $P = 0.006$) and *DQB1*0602* appears to be a candidate protective allele (RR = 0.26, $P = 0.02$) for TGC in the Japanese. None of the *HLA-DRB1* and *-DQB1* alleles showed a specific tendency for histological type or clinical stage of the tumours. Previous studies based on serotyping methods failed to show these allelic associations. High-resolution genotyping is essential because the peptide-binding domain of MHC class II molecules is determined more precisely by their genotypes than by their serotypes. In addition, inherent technical difficulties and typing errors of up to 25% make serotyping inefficient. Our results suggest that high-resolution genotyping is a useful genetic marker to determine risk for TGC.

**Keywords:** testicular germ cell carcinoma; MHC class II genotyping; modified PCR-RFLP; allele frequency

Testicular germ cell carcinoma (TGC), although relatively rare in the male population in general, is the most common malignancy in men between the ages of 15 and 35 years. Strong evidence for the involvement of genetic factors has been reported in TGC development. The incidence of TGC in first degree relatives is 2.2%, and in unrelated individuals 0.4%. For an individual who has a father or brother with TGC, the relative risk of developing TGC is increased sixfold compared with men in the general population. The concordance of tumour histology is high in twins and low in father–son pairs (Tolerud et al, 1985). Moreover, the incidence of TGC is relatively high among white American men in comparison with black American or Oriental men living in the same geographical areas (Senturia, 1987). Finally, as a pluripotential, polymorphic and commonly heterogeneous cancer, TGC is markedly affected by the host environment, e.g. when a murine embryonal carcinoma cell is placed in a mouse blastocyte, it may differentiate in an orderly fashion and participate in the creation of a normal mouse (Richie, 1992). All this evidence suggests that HLA association could be one of the genetic factors in the development of TGC.

Previous studies (DeWolf et al, 1979; Pollack et al, 1982; Oliver et al, 1986; Aiginger et al, 1987; Kratzig et al, 1989) on the association of HLA antigens with TGC have shown no association with the HLA-A and HLA-C regions, and inconsistent association with the HLA-B region. Despite the inherent technical limitations of conventional methods used for HLA-DR typing, such as cytotoxicity, previous studies have claimed a consistent association of TGC with HLA-DR antigens. Some of these studies have suggested the need for genotyping (Oliver et al, 1986; Yoshimura et al, 1993). Our study is the first high-resolution HLA-DRB1 and DQB1 genotyping of patients with TGC.

Reports published up to 1982 did not include HLA-DR. Later studies included the DR region, but the number of antigens determined was limited (Pollack et al, 1982). This means that, up to now, there has been no complete HLA-DRB1 typing, even by serotyping. The purpose of our molecular epidemiological study was to identify high-risk individuals, thereby allowing for their close surveillance and the early detection of these potentially curable tumours.

*Received 15 January 1997*
*Revised 15 April 1997*
*Accepted 1 May 1997*

*Correspondence to:* Osamu Yoshida, Department of Urology, Faculty of Medicine, Kyoto University, 54 Kawahara-cho, Shogoin, Sakyo-ku, Kyoto 606–01, Japan

### MATERIALS AND METHODS

### Patients and controls

Fifty-five Japanese patients with TGC confirmed histopathologically were obtained for study from the 1990–96 tumour registries at Kyoto University Hospital and its affiliated hospitals without any selection criteria. The histological diagnosis and clinical stage were based, respectively, on the modified WHO classification system (Mostofi and Sobin, 1977) and the tumour–nodes–metastasis staging systems (UICC, 1989). The clinicopathological profiles of the patients are given in Table 1. As controls, we used the genetic frequencies of HLA-DRB1 and -DQB1 in 1216 healthy Japanese subjects reported at the 11th Japanese HLA Genotyping Workshop held by the Japanese Society for Histocompatibility and Immunogenetics in 1994 (Akaza et al, 1994).

### DNA extraction and PCR-RFLP

High molecular weight genomic DNA was extracted from the patients' peripheral blood lymphocytes after proteinase K digestion by the standard phenol–chloroform method (Perucho et al, 1981). The modified PCR-RFLP method for HLA-DRB1 genotyping described by Ota et al (1992) and that for HLA-DQB1 described by Nomura et al (1991) were used. Briefly, the polymorphic exon 2

**Table 1.** Clinicopathological profiles of the TGC patients

| | |
|---|---|
| No. of patients | 55 |
| Age | 3–64 (35 ± 11)[a] |
| Histological type | |
| Pure seminoma | 31 |
| NSGCT[b] | 24 |
| TNM stage | |
| Low stage[c] | 34 |
| High stage[d] | 21 |

[a]Median ± SD. [b]Non-seminomatous germ cell tumours (with or without a seminomatous component). [c]N0, N1, N2A. [d]N2B, N3, M+.

domains of the *DRB1* and *DQB1* genes were amplified. Each PCR was amplified in a 25-μl mixture of approximately 100 ng of genomic DNA, 25 pmol of each primer, 500 μM each of dATP, dGTP, dCTP, and dTTP and 1.25 U of Ampli*Taq* DNA polymerase (Roche Molecular Systems, Branchburg, NJ, USA). This mixture was subjected to 35 PCR cycles, each of which was divided into periods of 94°C for 30 s, 55°C for 40 s, and 72°C for 60 s, with a final 3-min extension at 72°C. For some primers, the annealing temperature was increased to 62°C on the basis of preliminary PCR results in order to eliminate non-allelic bands. Loading buffer was mixed with 4 μl of the PCR products and electrophoresed in a 2% horizontal agarose gel in a minigel apparatus (Mupid-2, Cosmo Bio Co., Tokyo, Japan). Five microlitres of the PCR products was digested at the appropriate temperature for 1 h with 3–5 units of the following restriction enzymes: *Ava*II and *Pst*I for DRB1-DR1; *Fok*I, *Cfr*13I and *Hph*I for DRB1-DR2; *Sac*II, *Ava*II, *Hin*fI, *Hae*II, *Hph*I and *Mn*LI for DRB1-DR4; *Ava*II, *Fok*I, *Kpn*I, *Hae*II, *Cfr*13I, *Sfa*NI, *Sac*II, *Bsa*JI, *Apa*I and *Hph*I for DRB1-DR3568; FokI, ApaI, HaeII, SfaNI, BssHII and HphI for DQB1-DQ1; and FokI, BglI, SacI, AcyI and HpaII for DQB1-DQ2, 3, 4, together with the corresponding buffer (1–2 μl), at a final volume of 10 μl in distilled water. The digested PCR products were electrophoresed through a vertical polyacrylamide gel in a microgel apparatus then stained with ethidium bromide (0.5 mg ml$^{-1}$).

### Data analysis

Depending on the sample size, Fisher's exact probability test or the chi-square test was used to analyse all the 2 × 2 tables. $P < 0.05$ was considered statistically significant. Relative risk (RR) was calculated as $(a \times d)/(b \times c)$, where $a$, $b$, $c$ and $d$ are, respectively, the numbers of marker (+) patients, marker (–) patients, marker (+) controls and marker (–) controls. An *RR* value less than than 0.5 was assigned as relatively low risk, and an *RR* value more than 2 was assigned as relatively high risk. We performed significance tests only for those alleles with relatively low risk or relatively high risk. *P*-values were corrected by the number of comparisons made in the overall study. The δ-value was estimated according to Thompson (1981). Values of the preventive fractions (PFc, PF) and aetiological fractions (EFc, EF) were estimated according to Green (1982).

### RESULTS

The modified PCR-RFLP method produced accurate, reproducible, and easily discriminative cleavage patterns of high-resolution HLA-DRB1 and DQB1 genotyping (Fig. 1). The distribution of the HLA-DRB1 and DQB1 genotypes together with the

**Figure 1.** A representative 10% polyacrylamide/ethidium bromide-stained gel showing cleavage patterns of polymorphic restriction fragments obtained by the modified PCR-RFLP method from two patients with the protective allele, *DQB1*0602*. At completion of digestion, a coding system depending on the availability of digested (1), non-digested (0) and both digested and non-digested (2) bands was used. Left: The cleavage pattern of the protective allele, *DQB1*0602*, of patient A. Right: Its combination with the DQB1*0503 of patient B. *DQB1*0503* determination was clarified by further digestion with Hph I

*RR* ratios for 55 patients with TGC vs those for 1216 healthy Japanese control subjects are shown in Table 2. The HLA class II allele, *DRB1*0410*, a subtype of DR4, was significantly associated with susceptibility in Japanese TGC patients compared with the healthy Japanese control subjects (5.45% vs 1.79) (*RR* = 3.26, *P* = 0.006). The aetiological fraction of exposed individuals (EFc) carrying *DRB1*0410* was 0.69, which means hypothetically that, if the presence of this allele per se is a susceptibility factor for TGC development in Japanese male patients with TGC who carry the *DRB1*0410* allele, 69% of the cases developed because of the presence of this susceptibility gene. The aetiological fraction of the total population (EF) was 0.08, which means that 8% of Japanese male patients with TGC developed testicular cancer because of the presence of this susceptibility gene.

The association of the low relative risk values of the *DRB1*0406*, **1401* and *1501* and *DQB1*0502* alleles in patients with TGC was not significant when compared with the values for the healthy Japanese control subjects. *DQB1*0602* appears to be a candidate protective allele for patients with TGC compared with the healthy subjects (1.81% vs 6.22%) (*RR* = 0.26, *P* = 0.02). Because in our study this association was not significant after correction for the number of comparisons made, further studies are needed for confirmation. If the preventive fraction of individuals carrying *DQB1*0602* (PFc) was 0.74, which means that if *HLA-DQB1*0602* per se prevents TGC, then it would have prevented 74% of the cases that would otherwise have occurred among the *DQB1*0602*-positive individuals. The preventive fraction of the total population (PF)

© Cancer Research Campaign 1997

**Table 2.** Distribution of *HLA-DRB1* and *DQB1* alleles in 55 patients with TGC and 1216 healthy controls, and distribution of the histological type and TNM stage vs *HLA-DRB1* and *DQB1* alleles

| Alleles | TGC | Controls[a] | RR | *P* | Seminoma | NSGCT | TNM (low) | TNM (high) |
|---|---|---|---|---|---|---|---|---|
| *DRB1*0101* | 5 | 141 | 0.76 | – | 2 | 3 | 3 | 2 |
| **0301* | 0 | 2 | – | – | – | – | – | – |
| **0302* | 0 | 1 | – | – | – | – | – | – |
| **0401* | 0 | 15 | – | – | – | – | – | – |
| **0403* | 4 | 51 | 1.79 | – | 4 | 0 | 4 | 0 |
| **0404* | 0 | 11 | – | – | – | – | – | – |
| **0405* | 17 | 322 | 1.24 | – | 10 | 7 | 12 | 5 |
| **0406* | 1 | 74 | 0.28 | 0.15 | 0 | 1 | 0 | 1 |
| **0407* | 0 | 17 | – | – | – | – | – | – |
| **0408* | 0 | 3 | – | – | – | – | – | – |
| **0410* | 6 | 44 | 3.26 | 0.006 | 4 | 2 | 4 | 2 |
| **0700* | 1 | 6 | – | – | 1 | 0 | 1 | 0 |
| **0802* | 4 | 102 | 0.85 | – | 1 | 3 | 2 | 2 |
| **0803* | 10 | 202 | 1.12 | – | 7 | 3 | 5 | 5 |
| **0804* | 1 | 5 | – | – | 1 | 0 | 0 | 1 |
| **0901* | 14 | 342 | 0.87 | – | 8 | 6 | 9 | 5 |
| **1001* | 0 | 17 | – | – | – | – | – | – |
| **1101* | 3 | 63 | 1.05 | – | 2 | 1 | 2 | 1 |
| **1102* | 1 | 1 | – | – | 1 | 0 | 1 | 0 |
| **1103* | 2 | – | – | – | 1 | 1 | 1 | 1 |
| **1201* | 5 | 89 | 0.99 | – | 2 | 3 | 3 | 2 |
| **1202* | 3 | 43 | 1.57 | – | 3 | 0 | 2 | 1 |
| **1301* | 0 | 14 | – | – | – | – | – | – |
| **1302* | 8 | 166 | 1.07 | – | 2 | 6 | 5 | 3 |
| **1304* | 0 | 1 | – | – | – | – | – | – |
| **1401* | 1 | 82 | 0.25 | 0.11 | 0 | 1 | 0 | 1 |
| **1402* | 2 | 5 | – | – | 1 | 1 | 0 | 2 |
| **1403* | 0 | 46 | – | – | – | – | – | – |
| **1405* | 2 | 54 | 0.81 | – | 2 | 0 | 2 | 0 |
| **1406* | 1 | 42 | – | – | 1 | 0 | 1 | 0 |
| **1407* | 0 | 3 | – | – | – | – | – | – |
| **1501* | 4 | 173 | 0.47 | 0.09 | 2 | 2 | 2 | 2 |
| **1502* | 13 | 246 | 1.22 | – | 6 | 7 | 8 | 5 |
| **1601* | 0 | 1 | – | – | – | – | – | – |
| **1602* | 2 | 25 | – | – | 1 | 1 | 1 | 1 |
| *DQB1*0201* | 0 | 9 | – | – | – | – | – | – |
| **0301* | 12 | 282 | 0.92 | – | 6 | 6 | 7 | 5 |
| **0302* | 7 | 227 | 0.63 | – | 6 | 1 | 5 | 2 |
| **0303* | 19 | 361 | 1.25 | – | 10 | 9 | 12 | 7 |
| **0401* | 20 | 317 | 1.62 | – | 13 | 7 | 15 | 5 |
| **0402* | 6 | 97 | 1.41 | – | 4 | 2 | 3 | 3 |
| **0501* | 5 | 159 | 0.66 | – | 2 | 3 | 3 | 2 |
| **0502* | 1 | 61 | 0.35 | 0.210 | 1 | 0 | 1 | 0 |
| **0503* | 4 | 99 | 0.88 | – | 3 | 1 | 3 | 1 |
| **0504* | 0 | 1 | – | – | – | – | – | – |
| **0601* | 24 | 441 | 1.36 | – | 13 | 11 | 12 | 12 |
| **0602* | 2 | 151 | 0.26 | 0.020 | 1 | 1 | 1 | 1 |
| **0604–6* | 10 | 171 | 1.35 | – | 3 | 7 | 6 | 4 |

*RR*, relative risk; NSGCT, non-seminomatous germ cell tumours (with or without a seminomatous component).[a]Akaza et al (1994).

was 0.03. Correspondingly, *DQB1*0602* would have prevented 3% of the cases that would have developed among Japanese male individuals if this allele had not had a preventive effect.

Haplotype frequency and linkage disequilibrium of low-frequency alleles, *DRB1*1501;DQB1*0602*, in control subjects and patients were 6.16/3.64 and 1.21/2.95 respectively. The *DRB1*1501* and *DQB1*0602* haplotypes were less frequent and linkage disequilibrium was stronger in patients with TGC than in healthy control subjects. Haplotype frequency and linkage disequilibrium of high-frequency alleles, *DRB1*0410;DQB1*0402* in control subjects and patients were 1.54/7.27 and 4.05/22.45 respectively. The *DRB1*0410* and *DQB1*0402* haplotypes were more frequent and linkage disequilibrium was much stronger in patients with TGC than in healthy control subjects.

When more than one allele shows an association with a particular disease, the appropriate measure for determining which allele has the strongest association with the disease is the δ-value. The δ-value for the susceptibility allele *DRB1*0410* was 4.63, and for the relatively protective allele *DQB1*0602* was 0.85. The preventive fractions (PFc, PF) demonstrate that, among individuals who carry *DQB1*0602*, this allele is highly protective. In the general male population, expression of the *DRB1*0410* allele causes the highest susceptibility, and this allele is the allele most significantly associated with TGC tumours.

© Cancer Research Campaign 1997

The associations of *DRB1*0403* and *DQB1*0302* with seminoma and low-stage tumours were not significant ($P > 0.05$). There was no specific association between the other *DRB1* and *DQB1* alleles and histological type or clinical stage of the tumours. The clinicopathological data collected in our study were taken from first admission information, which may be the reason for the discrepancy. In fact, the status of both TGC patients who carried the protective allele *DQB1*0602*, one with initial stage pT2N0M0 seminoma and one with stage pT3N4M1 embryonal cell carcinoma, showed no evidence of disease after 5 years of survival.

### DISCUSSION

*HLA-DRB1*0410* showed a highly significant association with susceptibility to TGC, and *DQB1*0602* appeared to protect against TGC development. The validity of the latter statement, however, needs to be confirmed. Previous studies (DeWolf et al, 1979; Pollack et al, 1982; Oliver et al, 1986; Aiginger et al, 1987; Kratzig et al, 1989) that used conventional methods, such as microcytotoxicity, failed to show this allelic association. High-resolution genotyping is essential because the polymorphism of the peptide-binding domain of MHC class II molecules is more precisely determined by genotypes than by serotypes. The *HLA-DRB1* and *-DQB1* genes are extremely polymorphic, and the amino acids they encode are located in regions that line the sides and floor of the peptide-binding groove, which is related to immune responsiveness as well as to the ability to bind antigenic peptide and to be recognized by CD4 T-cell receptors (Rukstalis et al, 1989; Nomura et al, 1991; Ota et al, 1992; Ayala, 1995; Futami et al, 1995).

Despite the difficulties inherent in conventional HLA-typing methods, on the basis of NIH microcytotoxicity findings DeWolf et al (1979) reported that HLA-A, and -B antigens were not associated with TGC and that Drw7 was more frequent in teratocarcinoma (RR = 8.32). Pollack et al (1982), who typed 145 white American males with TGC tumours, found no association with HLA-C antigens, but reported a high frequency of DR5 in pure seminoma cases. Aiginger et al (1987), who typed 66 patients with TGC, have reported a high frequency of DR5 antigen with metastasis. Oliver et al (1986) have reported increased DR5 antigen and decreased DR3 antigen frequencies in seminoma. In 1989, Kratzig et al pooled all the previous data with their results on HLA typing for TGC and reported a significantly increased frequency of DR5 in seminoma and some association of HLA-B13 with non-seminomatous germ cell tumours. These previously reported findings are adequate for MHC class I typing but not for class II typing. In fact, none of the reports up to 1982 included HLA-DR and, although later studies included the DR region, the number of antigens determined was limited (Pollack et al, 1982). With regard to the serotype frequencies found in our study, DR5 was slightly more frequent in TGC patients than in the control subjects (12.72 vs 8.10) and showed a greater tendency for semonima. DR3 and DR7 are very infrequent in the Japanese population (0.12% and 0.25% respectively). The inconsistency in the reports on the association of some of HLA-B antigens with TGC tumours may stem from the strong linkage between the HLA-B and HLA-DRB1 regions. The weaker antigenicity of HLA-A than HLA-B reported in organ transplants, despite the antigens' structural similarities, is attributed mainly to the weak association of HLA-A with HLA-DR, whereas HLA-B has a strong association with HLA-DR (Tiercy et al, 1991; Ichikawa et al, 1993).

The strong influence of MHC complex genes on the development of diseases with a mainly autoimmune background has been reported. The demonstration of the major role of mouse MHC (H-2) in determining resistance to the development of murine leukaemia led to a number of studies. Two tumour types, Hodgkin's disease and nasopharyngeal carcinoma, have consistently been reported to be significantly associated with HLA antigens in both unrelated and familial studies (DeWolf et al, 1979; Oliver et al, 1986; Senturia, 1987).

Theoretically, cancer may arise under conditions of genetic or acquired reduced immune capacity. In patients undergoing organ transplants, a significant overall two- to five-fold increased risk of TGC tumours has been reported. This confirms that an impaired immune system allows carcinogenic factors to act. The worldwide epidemic of acquired immunodeficiency syndrome (AIDS) provides further evidence of the effect of and risk of specific cancers. Serotyping analyses showed that the development of Kaposi's sarcoma in patients with full-blown AIDS was strongly associated with HLA-DR5 (Pollack and Livingstone, 1985; Smeraldi et al, 1986; Birkeland et al, 1995).

An MHC class II allele, *DQB1*0301*, has been shown to be significantly linked to the incidence of melanoma and advanced disease status (Lee et al, 1994), indicative of the association of cancer development with MHC class II genes. Our findings that *HLA-DRB1*0101* and **0405* are protective alleles in patients with renal cell carcinoma (Özdemir et al, 1997) is well established, with spontaneous regression of the RCC in some patients. The association of cervical carcinoma and MHC class II genes is still a matter of dispute, mainly owing to typing difficulties as stated (Glew et al, 1992). In addition, because a greater number of class II genes have been detected by genomic analysis than by serology, new standards for bone marrow and other organ transplants have been established (Tiercy et al, 1991; Ichikawa et al, 1993).

In conclusion, genotyping of MHC class II genes promises to provide useful information for early tumour detection and for predicting the prognosis of patients with various malignancies.

### REFERENCES

Aiginger P, Kuzmits R, Kradzig C, Schwarz HP, Zielinski CC, Kühböck J and Mayr WR (1987) HLA antigens and germ cell tumours. *Lancet* **1**: 276–277

Akaza T, Imanishi T, Fujiwara K, Tokunaga K, Juji T, Yahiki S and Sonoda S (1994) HLA alleles and haplotype frequencies in Japanese. *Transplant Now* **7** (suppl.): 87–99

Ayala FJ (1995) The myth of Eve: molecular biology and human origins. *Science* **270**: 1930–1936

Birkeland SA, Storm HH, Lamm LU, Barlow L, Blohme I, Forsberg B, Eklund B, Fjeldborg O, Friedberg M, Frödin L, Glattre E, Halverson S, Holm NV, Jakobsen A, Jørgensen HE, Ladefoget J, Lindholm T, Lundgren G and Pukkala E (1995) Cancer risk after renal transplantation in Nordic countries, 1964–1986. *Int J Cancer* **60**: 183–189

DeWolf WC, Lange PH, Einarson ME and Yunis EJ (1979) HLA and testicular cancer. *Nature* **277**: 216–217

Futami S, Aoyama N, Honsako Y, Tamura T, Morimoto S, Nakashima T, Ohmoto A, Okano H, Miyamoto M, Inaba H, Naruse T, Nose Y and Kasuga M (1995) *HLA-DRB1*1502* allele, subtype of DR15, is associated with susceptibility to ulcerative colitis and its progression. *Dig Dis Sci* **40**: 814–818

Glew SS, Stern PS, Davidson JA and Dyer PA (1992) HLA antigens and cervical carcinoma. *Nature* **356**: 22

Green A (1982) The epidemiologic approach to studies of association between HLA and disease. *Tissue Antigens* **19**: 245–268

Ichikawa Y, Hashimoto M, Nojima M, Sata M, Fujimoto N, Kyo M, Ishibashi M, Ohshima S, Amemiya H, Fukunishi T, Nagano S and Sonoda T (1993) The significant effect of HLA-DRB1 matching on long-term kidney graft outcome. *Transplantation* **56**: 1368–1371

© Cancer Research Campaign 1997 

Kradzig C, Aiginger P, Kuzmits R, Spona J, Kirnbauer M, Seiser A and Mayr WR (1989) HLA-antigen distribution in seminoma, HCG-positive seminoma and non-seminomatous tumours of the testis. *Urol Res* **17**: 377–380

Lee JE, Reveille JD, Ross MI and Platsoucas CD (1994) *HLA-DQB*0301* association with increased cutaneous melanoma risk. *Int J Cancer* **59**: 510–513

Mostofi FK and Sobin LH (1977) Histological typing of testis tumours. In *International Histological Classification of Tumors*, No. 16. World Health Organization: Geneva

Nomura N, Ota M, Tsuji K and Inoko H (1991) HLA-DQB1 genotyping by a modified PCR-RFLP method combined with group-specific primers. *Tissue Antigens* **38**: 53–59

Oliver RTD, Stephenson CA, Parkinson MC, Forman D, Atkinson A, Bodmer J and Bodmer WF (1986) Germ cell tumors of the testicle as a model of MHC influence on human malignancy. *Lancet* **1**: 1506

Ota M, Seki T, Fukushima H, Tsuji K and Inoko H (1992) HLA-DRB1 genotyping by modified PCR-RFLP method combined with group-specific primers. *Tissue Antigens* **39**: 187–202

Özdemir E, Kakehi Y, Nakamura E, Kinoshita H, Terachi T, Okada Y and Yoshida O (1997) *HLA-DRB1*0101* and **0405* as protective alleles in Japanese patients with renal cell carcinoma. *Cancer Res* **57**: 742–746

Perucho M, Goldfarb M, Shimizu K, Lama C, Fogh J and Wigler M (1981) Human tumor-derived cell lines contain common and different transforming genes. *Cell* **27**: 467–476

Pollack MS and Livingstone IQ (1985) HLA and DR antigen frequencies in melanoma patients: possible relation to disease prognosis. *Tissue Antigens* **26**: 262–265

Pollack MS, Vugrin D, Hennessy W, Herr HW, Dupont B and Whitmore WF (1982) HLA antigens in patients with germ cell cancers of the testis. *Cancer Res* **42**: 2470–2473

Richie JP (1992) Neoplasms of the testis. In *Campbell's Urology*, Walsh PC, Retik AB, Stamey TA and Vaughan ED (eds), pp. 1222–1255. W.B. Saunders: Philadelphia

Rukstalis DB, Bubley GJ, Donahue JP, Richie JP, Seidman JG and DeWolf WC (1989) Regional loss of chromosome 6 in two urological malignancies. *Cancer Res* **49**: 5087–5090

Senturia YD (1987) The epidemiology of testicular cancer. *Br J Urol* **60**: 285–291

Smeraldi RS, Lazzarin A, Moroni M, Fabio G, Eisera NB and Zanussi C (1986) HLA-associated susceptibility to acquired immunodeficiency syndrome in Italian patients with human-immunodeficiency-virus infection. *Lancet* **2**: 1187–1189

Thomson G (1981) A review of theoretical aspects of HLA and disease associations. *Theor Popul Biol* **20**: 168–208

Tiercy JM, Morel J, Friedel AC, Zwahlen F, Gebuhrer L, Betuel H, Jeannet M and Mach B (1991) Selection of unrelated donors for bone marrow transplantation is improved by HLA class II genotyping with oligonucleotide hybridization. *Proc Natl Acad Sci USA* **88**: 7121–7125

Tolerud D, Blattner WA, Fraser MC, Brown LM, Pottern L, Shapiro E, Kirkemo A, Shawker TH, Jevadpour N, O'Connell K, Stutzman RE and Fraumeni JF (1985) Familial testicular cancer and urogenital developmental anomalies. *Cancer* **55**: 1849–1854

Union Internationale Contre Le Cancer (1989) *The TNM Classification of Malignant Tumours*, 3rd edn. Springer: Berlin

Yoshimura K, Nishimura K, Miyoshi S, Mizutani S, Mise T and Iwasa K (1993) Father–son testicular cancer. *Urol Int* **50**: 108–110

© Cancer Research Campaign 1997